# Communications on Hand

**Published:** 1875-06

**Authors:** 


*Communications on Hand.*

Accumulation and Inspissation of Cerumen, by Swan M. Burnett, M.D., Knoxville, Tenn.; An Easily Affected Method of Artificial Respiration, by J. B. Mattison; The Necessity of Active Treatment in all Acute Diseases of the Throat, by A. G. Smythe, M.D., Mississippi; Cincho Quinine, by A. G. Smythe, M.D., Mississippi; Cases in Practice: Post Partum Hemorrhage; Suspected Laceration of the Cervix Uteri—by F. K. Bailey, M.D., Knoxville, Tenn.; Old Eyes and Spectacles, by Swan M. Burnett, M.D., Knoxville, Tenn.; Clinical Notes, by F. K. Bailey, M.D., Knoxville, Tenn.; Five Cases of Operation by the Bloodless Method, by Lewis Balch, M.D., Albany, N. Y.; A Case of Albuminuric Eclampsia, by G. L. Ullman, M.D., Albany, N. Y.

